# Supplementary material for: An Overview of Ovarian Calyx Fluid Proteins of Toxoneuron nigriceps (Viereck) (Hymenoptera: Braconidae): An Integrated Transcriptomic and Proteomic Approach
Source: Biomolecules. 2023 Oct 19;13(10):1547. doi: 10.3390/biom13101547 (PMC10605793; doi:10.3390/biom13101547)
Supplement: Supplementary file 1 [file biomolecules-13-01547-s001.zip › Supplementary Table S2.pdf]

Line	Peptides	Contig	Frame n°	Sequence coverage	Name
**1**	**NO ID**				
**2**	31	T_C1643	3	35%	puromycin-sensitive aminopeptidase isoform X2 [Cotesia glomerata]
	29	T_C3890	2	23%	uncharacterized protein LOC103568231 [Microplitis demolitor]
**3**	48	T_C141	5	75%	transferrin [Cotesia flavipes]
	26	T_C2568	5	34%	dipeptidyl peptidase 3 isoform X1 [Microplitis demolitor]
	21	T_C2767	2	34%	putative prolyl endopeptidase [Cotesia chilonis]
	11	T_C1743	4	15%	Cytoplasmic aconitate hydratase [Cotesia chilonis]
	8	T_C241	3	12%	replication factor C subunit 1 [Microplitis demolitor]
**4**	26	T_C387	1	48%	uncharacterized protein LOC103580511 isoform X2 [Microplitis demolitor]
	23	T_C33	6	41%	putative heat shock 70 kDa protein cognate 4 [Cotesia chilonis]
	16	T_C216	3	23%	uncharacterized protein LOC103580511 isoform X2 [Microplitis demolitor]
	13	T_C1002	4	33%	hypothetical protein G9C98_004494 [Cotesia typhae]
	9	T_C2350	6	23%	60 kDa heat shock protein, mitochondrial-like [Fopius arisanus]
	8	T_C1259	2	12%	uncharacterized protein LOC103580511 isoform X1 [Microplitis demolitor]
	5	T_C1206	4	13%	putative envelope protein ODV-E66-2 [Microplitis demolitor]
**5**	21	T_C1221	3	48%	cytosol aminopeptidase [Cotesia chilonis]
	13	T_C2380	6	35%	putative aldeìhyde dehydrogenase, mitochondrial [Cotesia chilonis]
	15	T_C2225	5	28%	protein disulfide-isomerase [Microplitis demolitor]
	9	T_C1074	1	18%	toll-like receptor 13 [Microplitis demolitor]
	8	T_C3830	1	21%	retinoid-inducible serine carboxypeptidase-like [Cotesia glomerata]
	5	T_C351	3	11%	trehalase-like [Microplitis demolitor]
	4	T_C2989	2	10%	A disintegrin and metalloproteinase with thrombospondin motifs 1-like [Microplitis demolitor]

**6**	24	T_C116	1	51%	FK506-binding protein 59 isoform X1 [Microplitis demolitor]
	13	T_C1266	4	35%	rab GDP dissociation inhibitor alpha [Microplitis demolitor]
	4	T_C2385	5	11%	alpha-N-acetylgalactosaminidase [Cotesia glomerata]
	4	T_C3206	4	10%	esterase FE4-like [Chelonus insularis]
	10	T_C2581	3	22%	putative adenosylhomocysteinase [Cotesia chilonis]
	17	T_C376	6	40%	putative phosphoglycerate kinase [Cotesia chilonis]
**7**	25	T_C1316	5	60%	arginine kinase [Microplitis demolitor]
	13	T_C271	6	43%	uncharacterized protein LOC103572160 [Microplitis demolitor]
	10	T_C3785	4	27%	uncharacterized protein LOC103574027 [Microplitis demolitor]
	10	T_C3024	2	50%	leukocyte elastase inhibitor-like isoform X3 [Cotesia glomerata]
	10	T_C2661	1	21%	putative malate dehydrogenase, cytoplasmic [Cotesia chilonis]
	7	T_C38	4	19%	enolase isoform X2 [Microplitis demolitor]
	8	T_C2407	6	18%	putative uncharacterized protein DDB_G0286901 [Microplitis demolitor]
	4	T_C3680	2	11%	uncharacterized protein Vlf-1 [Microplitis demolitor]
	4	T_C3228	3	15%	putative inorganic pyrophosphatase [Cotesia chilonis]
	3	T_C127	4	14%	calcineurin-binding protein cabin-1 [Microplitis demolitor]
**8**	21	T_C1057	6	53%	aldose reductase [Microplitis demolitor]
	15	T_C3197	3	47%	aldo-keto reductase family 1 member B1-like [Cotesia glomerata]
	15	T_C1145	4	47%	UDP-glucose 4-epimerase isoform X4 [Diachasma alloeum]
	11	T_C1283	2	37%	uncharacterized protein Vp39 [Microplitis demolitor]
	13	T_C118	4	28%	eukaryotic translation initiation factor 3 subunit M isoform X2 [Microplitis demolitor]
	8	T_C1629	1	19%	major royal jelly protein 5 [Microplitis demolitor]
	5	T_C1800	2	18%	coiled-coil domain-containing protein 186 [Microplitis demolitor]
	6	T_C1239	2	13%	inositol-3-phosphate synthase 1-B [Cotesia glomerata]

	4	T_C1593	3	12%	DNA-(apurinic or apyrimidinic site) endonuclease [Cotesia glomerata]
	8	T_C2695	1	28%	glyoxalase domain-containing protein 4 [Microplitis demolitor]
	7	T_C205	4	21%	lysosomal aspartic protease-like [Microplitis demolitor]
	5	T_C1125	6	13%	chitinase-like protein Idgf4 [Microplitis demolitor]
	4	T_C1343	6	14%	Actin, muscle [Eumeta japonica]
	4	T_C1227	1	12%	serpin B6 isoform X2 [Microplitis demolitor]
	4	T_C1333	4	16%	putative ester hydrolase C11orf54 homolog [Cotesia chilonis]
	5	T_C1672	5	13%	uncharacterized protein LOC103568677 [Microplitis demolitor]
	4	T_C2877	6	11%	protein phosphatase 1 regulatory subunit 7 [Cotesia glomerata]
**9**	13	T_C242	2	39%	putative 14-3-3 protein zeta isoform X1 [Cotesia chilonis]
	19	T_C2966	3	46%	phosphoglycerate mutase 2 [Cotesia glomerata]
	7	T_C1460	5	40%	dihydropteridine reductase [Chelonus insularis]
	6	T_C262	5	22%	dr1-associated corepressor homolog [Microplitis demolitor]
	8	T_C2742	5	30%	lipoyl synthase, mitochondrial [Cotesia glomerata]
	8	T_C3314	4	19%	cytosolic non-specific dipeptidase [Microplitis demolitor]
	8	T_C2943	6	31%	hydroxyacylglutathione hydrolase, mitochondrial [Microplitis demolitor]
	5	T_C1175	2	13%	threonine--tRNA ligase, cytoplasmic isoform X2 [Microplitis demolitor]
	5	T_C2669	4	25%	putative envelope protein ODV-E66-17 [Microplitis demolitor]
	3	T_C3720	1	14%	S-methyl-5'-thioadenosine phosphorylase [Cotesia glomerata]
	4	T_C1759	1	14%	proteasome subunit alpha type-3 [Microplitis demolitor]
	3	T_C338	2	10%	Eukaryotic translation initiation factor 3 subunit I [Cotesia chilonis]
	3	T_C290	5	15%	proteasome subunit alpha type-7-1 [Diachasma alloeum]
	3	T_C3107	4	10%	eukaryotic translation initiation factor 3 subunit I [Microplitis demolitor]
	5	T_C2496	5	22%	phosphatidylinositol transfer protein alpha isoform isoform X1 [Microplitis demolitor]

	4	T_C1536	3	10%	putative mitochondrial complement component 1 Q subcomponent-binding protein-like protein [Cotesia chilonis]
	3	T_C346	4	12%	ribonuclease Oy [Cotesia glomerata]
	2	T_C3087	2	13%	proteasome subunit alpha type-4 [Chelonus insularis]
	2	T_C2601	5	10%	26S proteasome non-ATPase regulatory subunit 10 [Microplitis demolitor]
**10**	15	T_C2043	1	51%	glutathione S-transferase [Microplitis demolitor]
	8	T_C2091	2	15%	stress-induced-phosphoprotein 1 [Microplitis demolitor]
	9	T_C2739	5	36%	Krueppel homolog 1-like [Cotesia glomerata]
	6	T_C220	2	20%	glutathione S-transferase-like [Cotesia glomerata]
	6	T_C2055	1	24%	putative ferritin heavy polypeptide-like 17 isoform X1 [Cotesia chilonis]
**11**	12	T_C1492	6	36%	heat shock 70 kDa protein 4 [Microplitis demolitor]
	9	T_C333	6	45%	protein D2 isoform X2 [Microplitis demolitor]
	5	T_C1929	4	13%	uncharacterized protein 35a-6 [Microplitis demolitor]
	4	T_C1184	4	15%	proteasome subunit beta type-2 [Microplitis demolitor]
	3	T_C3115	5	12%	proteasome subunit beta type-6 [Microplitis demolitor]
	5	T_C1171	3	18%	dymeclin isoform X2 [Cotesia glomerata]
	2	T_C3284	1	11%	26S proteasome non-ATPase regulatory subunit 7 [Microplitis demolitor]
**12**	15	T_C201	5	54%	glyceraldehyde-3-phosphate dehydrogenase [Microplitis demolitor]
	7	T_C367	2	35%	putative peroxiredoxin 1 [Cotesia chilonis]
	7	T_C1185	5	48%	superoxide dismutase [Cu-Zn] [Microplitis demolitor]
	7	T_C154	5	33%	peptidyl-prolyl cis-trans isomerase-like [Microplitis demolitor]
	5	T_C2290	6	25%	peroxiredoxin-5, mitochondrial [Cotesia glomerata]
	2	T_C1443	4	16%	replication factor C subunit 3 [Microplitis demolitor]
	3	T_C2276	3	11%	programmed cell death protein 6 isoform X1 [Cotesia glomerata]
	4	T_C10718	1	35%	neprilysin-11-like [Microplitis demolitor]
	3	T_C1271	1	24%	calmodulin [Aeromonas veronii]

	2	T_C12915	3	20%	U-scoloptoxin(19)-Sm1a [Chelonus insularis]
**13**	8	T_C3625	6	74%	glutaredoxin-C4 isoform X1 [Microplitis demolitor]
	6	T_C1735	3	11%	replication protein A 70 kDa DNA-binding subunit [Microplitis demolitor]
	6	T_C102	3	12%	elongation factor 1-gamma [Microplitis demolitor]
	4	T_C3432	6	13%	eukaryotic translation initiation factor 2 subunit 1 [Cotesia glomerata]
	7	T_C349	6	26%	Similar to PCNA: Proliferating cell nuclear antigen [Cotesia congregata]
	6	T_C1034	1	33%	uncharacterized protein LOC118066271 [Chelonus insularis]
	4(3)	T_C1866	3	36%	activated RNA polymerase II transcriptional coactivator p15 [Microplitis demolitor]
	3(2)	T_C3427	5	16%	myophilin isoform X1 [Microplitis demolitor]
	2(2)	T_C1835	1	17%	cytochrome c [Osmia bicornis bicornis]
	2(2)	T_C1534	4	13%	SEC14-like protein 2 [Microplitis demolitor]
