# Supplementary material for: An Overview of Ovarian Calyx Fluid Proteins of Toxoneuron nigriceps (Viereck) (Hymenoptera: Braconidae): An Integrated Transcriptomic and Proteomic Approach
Source: Biomolecules. 2023 Oct 19;13(10):1547. doi: 10.3390/biom13101547 (PMC10605793; doi:10.3390/biom13101547)
Supplement: Supplementary file 1 [file biomolecules-13-01547-s001.zip › Supplementary Table S4.pdf]

**N.**	**Contig code**	**Functional annotatio**	**ORF**
1	T_C1643	puromycin-sensitive aminopeptidase isoform X2 [Cotesia glomerata]	CLOSED
2	T_C33	putative heat shock 70 kDa protein cognate 4 [Cotesia chilonis]	CLOSED
3	T_C1002	hypothetical protein G9C98_004494 [Cotesia typhae]	CLOSED
4	T_C2350	60 kDa heat shock protein, mitochondrial-like [Fopius arisanus]	CLOSED
5	T_C2380	putative aldeìhyde dehydrogenase, mitochondrial [Cotesia chilonis]	OPEN
6	T_C2989	A disintegrin and metalloproteinase with thrombospondin motifs 1-like [Microplitis demolitor]	OPEN
7	T_C116	FK506-binding protein 59 isoform X1 [Microplitis demolitor]	OPEN
8	T_C1266	rab GDP dissociation inhibitor alpha [Microplitis demolitor]	CLOSED
9	T_C376	putative phosphoglycerate kinase [Cotesia chilonis]	CLOSED
10	T_C1316	arginine kinase [Microplitis demolitor]	CLOSED
11	T_C2661	putative malate dehydrogenase, cytoplasmic [Cotesia chilonis]	OPEN
12	T_C38	enolase isoform X2 [Microplitis demolitor]	OPEN
13	T_C1057	aldose reductase [Microplitis demolitor]	OPEN
14	T_C3197	aldo-keto reductase family 1 member B1-like [Cotesia glomerata]	CLOSED
15	T_C1145	UDP-glucose 4-epimerase isoform X4 [Diachasma alloeum]	OPEN
16	T_C118	eukaryotic translation initiation factor 3 subunit M isoform X2 [Microplitis demolitor]	CLOSED
17	T_C1239	inositol-3-phosphate synthase 1-B [Cotesia glomerata]	OPEN
18	T_C1593	DNA-(apurinic or apyrimidinic site)	OPEN

		endonuclease [Cotesia glomerata]	
19	T_C2695	glyoxalase domain-containing protein 4 [Microplitis demolitor]	OPEN
20	T_C1343	Actin, muscle [Eumeta japonica]	CLOSED
21	T_C1333	putative ester hydrolase C11orf54 homolog [Cotesia chilonis]	CLOSED
22	T_C2877	protein phosphatase 1 regulatory subunit 7 [Cotesia glomerata]	CLOSED
23	T_C242	putative 14-3-3 protein zeta isoform X1 [Cotesia chilonis]	OPEN
24	T_C2966	phosphoglycerate mutase 2 [Cotesia glomerata]	OPEN
25	T_C1460	dihydropteridine reductase [Chelonus insularis]	CLOSED
26	T_C262	dr1-associated corepressor homolog [Microplitis demolitor]	OPEN
27	T_C2742	lipoyl synthase, mitochondrial [Cotesia glomerata]	OPEN
28	T_C3314	cytosolic non-specific dipeptidase [Microplitis demolitor]	OPEN
29	T_C2943	hydroxyacylglutathione hydrolase, mitochondrial [Microplitis demolitor]	OPEN
30	T_C1759	threonine--tRNA ligase, cytoplasmic isoform X2 [Microplitis demolitor]	CLOSED
31	T_C1759	proteasome subunit alpha type-3 [Microplitis demolitor]	OPEN
32	T_C338	Eukaryotic translation initiation factor 3 subunit I [Cotesia chilonis]	OPEN
33	T_C290	proteasome subunit alpha type-7-1 [Diachasma alloeum]	OPEN
34	T_C3107	eukaryotic translation initiation factor 3 subunit I [Microplitis demolitor]	CLOSED
35	T_C2496	phosphatidylinositol transfer protein alpha isoform isoform X1 [Microplitis demolitor]	CLOSED
36	T_C1536	putative mitochondrial complement component 1 Q subcomponent-binding protein-like protein [Cotesia chilonis]	OPEN

37	T_C3087	proteasome subunit alpha type-4 [Chelonus insularis]	OPEN
38	T_C2601	26S proteasome non-ATPase regulatory subunit 10 [Microplitis demolitor]	OPEN
39	T_C2043	glutathione S-transferase [Microplitis demolitor]	OPEN
40	T_C220	glutathione S-transferase-like [Cotesia glomerata]	OPEN
41	T_C1492	heat shock 70 kDa protein 4 [Microplitis demolitor]	CLOSED
42	T_C333	protein D2 isoform X2 [Microplitis demolitor]	OPEN
43	T_C1184	proteasome subunit beta type-2 [Microplitis demolitor]	OPEN
44	T_C3115	proteasome subunit beta type-6 [Microplitis demolitor]	OPEN
45	T_C3284	26S proteasome non-ATPase regulatory subunit 7 [Microplitis demolitor]	CLOSED
46	T_C201	glyceraldehyde-3-phosphate dehydrogenase [Microplitis demolitor]	OPEN
47	T_C2290	peroxiredoxin-5, mitochondrial [Cotesia glomerata]	CLOSED
48	T_C1443	replication factor C subunit 3 [Microplitis demolitor]	OPEN
49	T_C102	elongation factor 1-gamma [Microplitis demolitor]	CLOSED
50	T_C349	Similar to PCNA: Proliferating cell nuclear antigen [Cotesia congregata]	OPEN
51	T_C1866	activated RNA polymerase II transcriptional coactivator p15 [Microplitis demolitor]	OPEN
52	T_C3427	myophilin isoform X1 [Microplitis demolitor]	OPEN
53	T_C1835	cytochrome c [Osmia bicornis bicornis]	OPEN
54	T_C1534	SEC14-like protein 2 [Microplitis demolitor]	OPEN
