# Supplementary figures and images for: An Overview of Ovarian Calyx Fluid Proteins of Toxoneuron nigriceps (Viereck) (Hymenoptera: Braconidae): An Integrated Transcriptomic and Proteomic Approach
Source: Biomolecules. 2023 Oct 19;13(10):1547. doi: 10.3390/biom13101547 (PMC10605793; doi:10.3390/biom13101547)

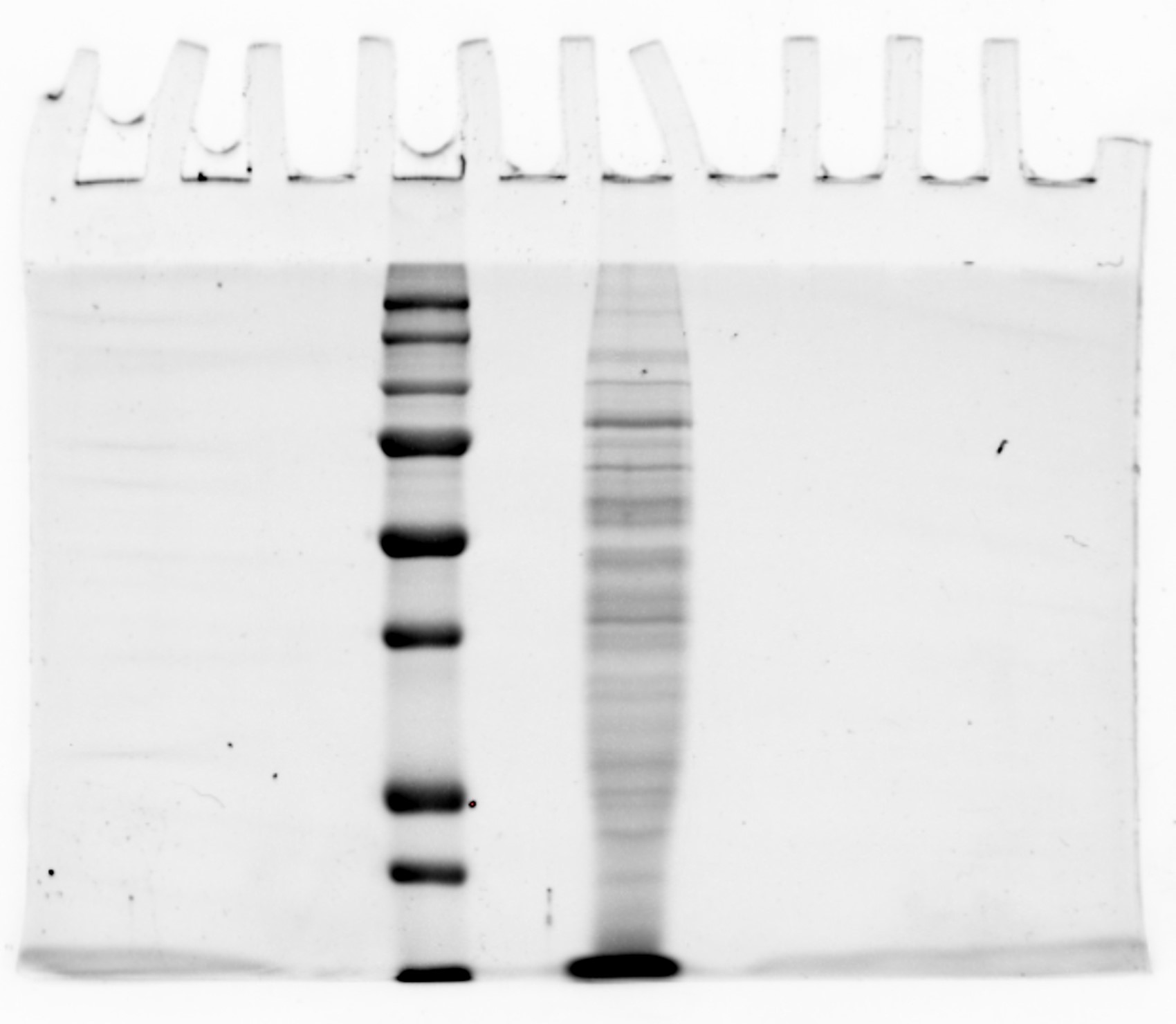

Supplement: Supplementary file 1 [file biomolecules-13-01547-s001.zip › Figure S1.jpg]
